# The Bactericidal Activity of Temporin Analogues Against Methicillin Resistant *Staphylococcus aureus*

**DOI:** 10.3390/ijms20194761

**Published:** 2019-09-25

**Authors:** Anna Golda, Paulina Kosikowska-Adamus, Aleksandra Kret, Olena Babyak, Kinga Wójcik, Ewelina Dobosz, Jan Potempa, Adam Lesner, Joanna Koziel

**Affiliations:** 1 Department of Microbiology, Faculty of Biochemistry, Biophysics and Biotechnology, Jagiellonian University, 30-387 Krakow, Poland; anna.b.golda@uj.edu.pl (A.G.); ola.kret57@gmail.com (A.K.); olenka.babyak@gmail.com (O.B.); kinga.wojcik@uj.edu.pl (K.W.); ewelina.blazusiak@uj.edu.pl (E.D.); jspote01@louisville.edu (J.P.); 2 Faculty of Chemistry, University of Gdansk, 80-309 Gdansk, Poland; paulina.kosikowska-adamus@ug.edu.pl (P.K.-A.); adam.lesner@ug.edu.pl (A.L.); 3 Department of Oral Immunity and Infectious Diseases, University of Louisville School of Dentistry, Louisville, KY 40202, USA

**Keywords:** temporin, MRSA, antimicrobial peptide, human keratinocytes

## Abstract

*Staphylococcus aureus* is a major infectious agent responsible for a plethora of superficial skin infections and systemic diseases, including endocarditis and septic arthritis. Recent epidemiological data revealed the emergence of resistance to commonly used antibiotics, including increased numbers of both hospital- and community-acquired methicillin-resistant *S. aureus* (MRSA). Due to their potent antimicrobial functions, low potential to develop resistance, and immunogenicity, antimicrobial peptides (AMPs) are a promising alternative treatment for multidrug-resistant strains. Here, we examined the activity of a lysine-rich derivative of amphibian temporin-1CEb (DK5) conjugated to peptides that exert pro-proliferative and/or cytoprotective activity. Analysis of a library of synthetic peptides to identify those with antibacterial potential revealed that the most potent agent against multidrug-resistant *S. aureus* was a conjugate of a temporin analogue with the synthetic Leu-enkephalin analogue dalargin (DAL). DAL-PEG-DK5 exerted direct bactericidal effects via bacterial membrane disruption, leading to eradication of both planktonic and biofilm-associated staphylococci. Finally, we showed that accumulation of the peptide in the cytoplasm of human keratinocytes led to a marked clearance of intracellular MRSA, resulting in cytoprotection against invading bacteria. Collectively, the data showed that DAL-PEG-DK5 might be a potent antimicrobial agent for treatment of staphylococcal skin infections.

## 1. Introduction

Recent epidemiological and clinical data indicate that infectious diseases are a fast-growing health concern. Overuse of antibiotics in humans and animals contributes to the selection of a variety of pathogens that are resistant to conventional drugs. *Staphylococcus aureus* has the ability to become resistant to nearly all clinically useful antibiotics through the development of resistance mechanisms acquired by horizontal transfer and by chromosomal mutation [1]. Methicillin-resistant *S. aureus* (MRSA) was reported for the first time among nosocomial isolates in 1961 [2]. Up until the 1990s, MRSA was restricted to hospitals and intensive care units, after which novel strains of MRSA emerged in the community [3]. At present, MRSA is one of the most common antibiotic-resistant *S. aureus* strains that cause serious, often detrimental, infections in many parts of the world, including Europe, the Americas, North Africa, the Middle East, and East Asia [4]. Indeed, most nosocomial staphylococcal infections in intensive care units (40–70%) are caused by MRSA strains [3,5,6,7]. Skin and soft tissue infections (SSTIs), severe necrotizing pneumonia, and bacteremia are the most common clinical presentations strongly associated with MRSA [3,7,8]. The rise of MRSA and other multidrug-resistant (MDR) staphylococci is a serious problem with respect to treatment and control. Therefore, there is an urgent need to develop new therapeutics. Among candidates are host defense peptides, also called antimicrobial peptides (AMPs), which are part of the natural defenses of all phyla of multicellular organisms, including plants, insects, amphibians, and mammals [9,10]. Most AMPs are small cationic molecules that act as potent antimicrobial compounds against a broad spectrum of microorganisms [11].

Amphibian skin granular glands are one of the richest sources of AMPs, especially those of the *Rana* genus, a widely distributed group with over 250 species worldwide [12]. Among the AMP families identified in frog skin are temporins, which are short (10–14 amino acids), hydrophobic, C-terminally amidated AMPs [13]. Temporins act as potent AMPs against staphylococci [14,15,16]. Here, we focused on temporin-1CEb, a naturally occurring 12-residue peptide derived from frog skin secretions that display antimicrobial activity against a broad spectrum of gram-positive and (albeit to a lesser extent) gram-negative bacteria [17,18]. Moreover, temporins, including temporin-1CEb, play a significant anti-inflammatory role both in vitro and in vivo [19]. The emergence of MRSA has resulted in an epidemic of SSTIs; thus, we created a library of compounds based on an analogue of temporin-1CEb (DK5) conjugated to peptides showing cytoprotective, pro-proliferative, and pro-migratory effects against human keratinocytes (cells that facilitate wound-healing) [20,21,22,23,24]. All selected peptides, including carnosine (CAR), dalargin (DAL), and COMB1, were linked to DK5 by a short biologically inert and non-immunogenic PEG linker (8-amino-3,6-dioxaoctanoic acid) [25].

We investigated the antibacterial activity of the designed peptides against different clinical isolates of MDR *S. aureus*. The data revealed the unique properties of DAL-PEG-DK5, which inhibited the growth of all tested MRSA strains, including planktonic and biofilm bacteria. We also assessed the mechanism by which DAL-PEG-DK5 disrupts the cytoplasmic membrane of staphylococci and evaluated the antibacterial activity of the conjugate against MRSA-infected human keratinocytes. Importantly, the data strongly support the potential of DAL-PEG-DK5 as a topical agent for treatment of MRSA.

## 2. Results

### 2.1. Antibacterial Activity of Temporin Conjugates Against Staphylococci

The antimicrobial activity of peptide conjugates (Table S1) was tested against staphylococci—*S. aureus* (Newman, ATCC 25923, USA300) and *S. epidermidis* (ATCC 12228). A broth microdilution method was used to determine the MIC (minimal inhibitory concentration) as a standard protocol. According to the obtained results (Table 1), the strongest bacteriostatic activity against all tested strains was observed for DAL-PEG-DK5 (MIC of 40 μg/mL against *S. aureus* USA300 and ATCC 25923, and 60 μg/mL against *S. epidermidis*). The only exception was the Newman strain, for which the MIC was 140 μg/mL. Moreover, CAR3-PEG-DK5, CAR-PEG-DK5, and COMB-1-PEG-DK5 did not inhibit growth of tested strains at concentrations up to 190 μg/mL.

Based on the above results, we selected DAL-PEG-DK5 for further studies to examine how the composition of this conjugate can influence its bacteriostatic effects. Thus, we measured the MIC of native peptides (DAL and DK5), two peptide conjugates (DAL-PEG-DK5 and DK5-PEG-DAL), and a scramble peptide (SCR). We tested these against the MRSA strain *S. aureus* USA300. MIC analysis revealed that neither the native peptides DK5 and DAL, nor the scramble peptide SRC, inhibited bacterial growth (Table 2).

Notably, DK5-PEG-DAL inhibited USA300 growth less effectively (four times less) than its sister analogue with the reversed connection between three components of DAL-PEG-DK5. The MIC values for DAL-PEG-DK5 and DK5-PEG-DAL against USA300 were 40 and 160 μg/mL, respectively. These results indicate that the DK5 conjugate shows potential antibacterial activity when DAL is localized at the C-terminus of the molecule.

Because USA300 was highly sensitive to the test conjugate, we decided to introduce other MRSA *S. aureus* strains. The majority of these strains (11/13) were isolated from the blood of infected patients, whereas two were obtained from skin. All strains were characterized (Table S2) before their sensitivity to DAL-PEG-DK5 was tested. The MIC assay revealed that all were highly sensitive to DAL-PEG-DK5. For 84% of the tested strains (11/13), the MIC was ≤ 40 μg/mL (Table 3). Taken together, the results show that DAL-PEG-DK5 inhibits the growth of different MRSA strains.

**Figure 1:**
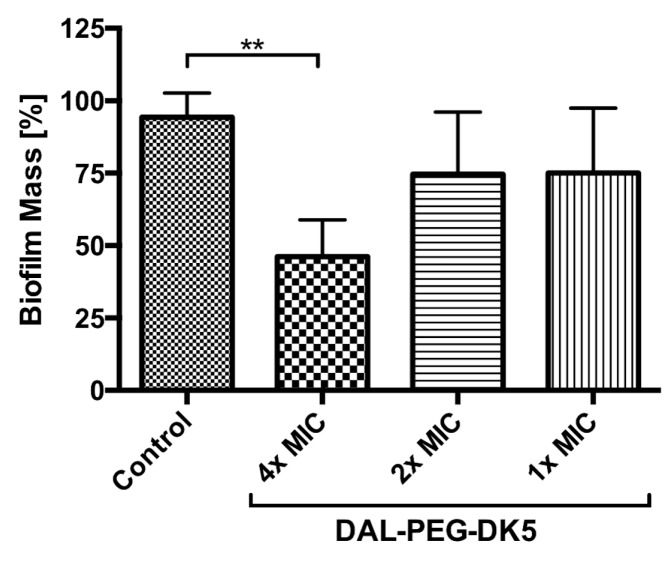
The effect of DAL-PEG-DK5 on the biofilm formed by *S. aureus*. USA300 strain was grown in 96-well plates at 37 °C for 24 h to form biofilm. Peptide was added at desired concentrations and plates were incubated for additional 24 h. After incubation with DAL-PEG-DK5, the biofilm was stained with crystal violet, then the dye was extracted with ethanol, measured OD and presented as percentage of biofilm reduction compared to untreated wells (Control). Mean ± SD *n* = 3. ** *p* < 0.0151; one-way ANOVA.

Biofilm formation is one of strategies used by *S. aureus* to compromise host responses and protect the bacteria from antibiotic and antiseptics agents. Currently, there are no antimicrobials that treat biofilm infections specifically [26]; thus, we aimed to examine whether DAL-PEG-DK5 disrupts mature biofilms (formed after 24 h) of USA300. As shown in Figure 1, the peptide conjugate (at 4 × MIC, equivalent to 160 μg/mL) disrupted the mature biofilm of MRSA, reducing biofilm mass by 50%.

Because the conjugate of DK5 with dalargin exhibited strong antibacterial activity against MRSA and efficiently reduced biofilm formation by staphylococci, we used this compound in all further experiments.

### 2.2. Mechanism Underlying the Bactericidal Activity of DAL-PEG-DK5

**Figure 2:**
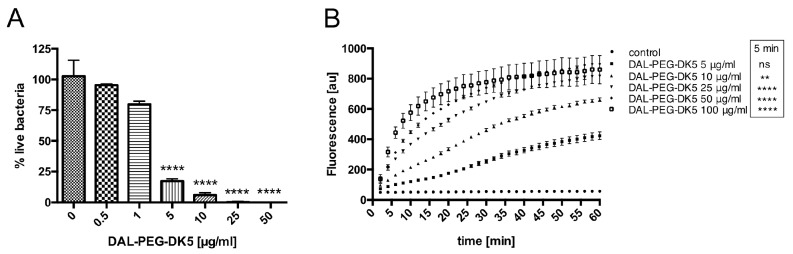
Bactericidal effect of DAL-PEG-DK5 on *S. aureus*. (**A**) SYTO 9 and PI staining. Staphylococci were treated with DAL-PEG-DK5 (0.5–50 μg/mL) for 3 h and stained with SYTO 9 (all bacteria in the population) and PI (bacteria with damaged membrane). The fluorescence was measured (excitation at 485 nm and emission at 530 nm (SYTO9) and 630 nm (PI)). Mean ± SD *n* = 2. **** *p* < 0.0001; one-way ANOVA. (**B**) Time dependent influx of Sytox green into USA300. Staphylococci were incubated with 1 μM Sytox green for 15 min and then DAL-PEG-DK5 was added (5–100 μg/mL). Probes were incubated 60 min and fluorescence was measured at indicated time points (excitation at 485 nm and emission at 520 nm). The data shown is representative of three separate experiments performed in triplicate. Mean ± SD. ns – non-significant, ** *p* < 0.005; **** *p* < 0.0001; one-way ANOVA.

The ability of DAL-PEG-DK5 to inhibit the growth of staphylococci and disrupt the mature biofilms formed by *S. aureus* suggests that the peptide also exerts bactericidal activity. To verify this hypothesis, we used LIVE/DEAD staining with DNA-binding dyes SYTO9 and propidium iodide (PI). SYTO9 is a membrane permeable (green) DNA stain that labels all bacteria in a population (those with intact membranes and those with damaged membranes). By contrast, PI (red) penetrates only bacteria with damaged membranes, causing a reduction in SYTO9 fluorescence when both dyes are present. Using this approach, we counted the total number of bacteria (green) and the number of non-viable bacteria (red). *S. aureus* USA300 was incubated with different concentrations of DAL-PEG-DK5 (0.5–50 μg/mL) for 3 h, and then stained with SYTO9 and PI (Figure 2A). We observed 20% and 5% live bacteria after exposure to 5 and 10 μg/mL, respectively, DAL-PEG-DK5.

Next, to explore the mechanism underlying DAL-PEG-DK5-mediated killing of MRSA, we examined the disruption of the cytoplasmic membrane of USA300 cells loaded with 1 μM Sytox green. The fluorescent dye does not cross intact membranes; thus, there is no change in intracellular Sytox green fluorescence without membrane disruption. When the peptide conjugate was added to USA300, we observed a dose-dependent increase in fluorescence intensity (reflecting bacterial membrane disruption) after only 5 min (Figure 2B). Moreover, at a concentration of 25 μg/mL peptide, permeabilization of the bacterial membrane reached a plateau. Taken together, these data suggest that DAL-PEG-DK5 exerts bactericidal effects against *S. aureus* MRSA strains by disrupting the bacterial cell membrane.

### 2.3. Activity of DAL-PEG-DK5 Against Intracellular S. aureus

For many years, *S. aureus* was considered an extracellular pathogen; however, recent data indicate that the bacteria can invade and persist within a variety of mammalian cells, including epithelial cells and even professional phagocytes [27,28]. One type of host cell that is efficiently colonized by *S. aureus* is the keratinocyte [29]. This strategy of staphylococci seems to be important for chronic and recurrent skin infections [30,31]. Therefore, it is important to evaluate the intracellular activity of DAL-PEG-DK5 against *S. aureus* that colonize the cytoplasm of human keratinocytes.

**Figure 3:**
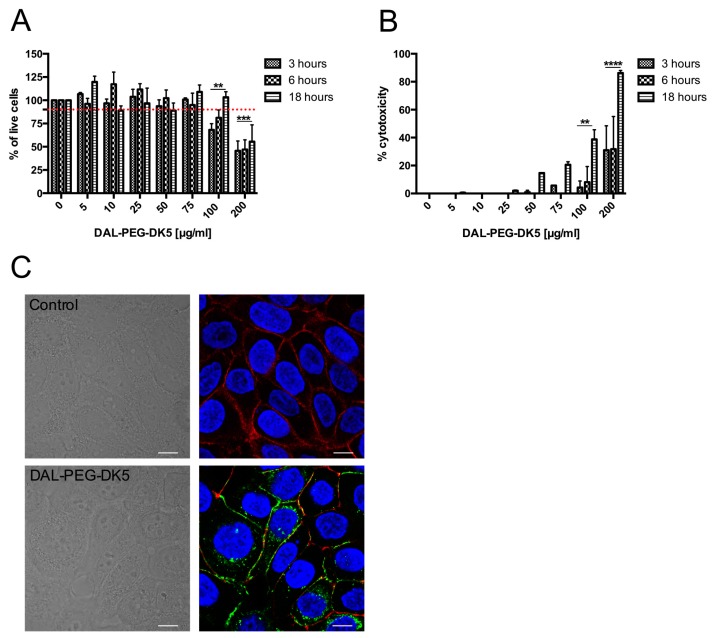
The influence of DAL-PEG-DK5 on physiology of human keratinocytes. The potentially toxic effect of DAL-PEG-DK5 on HaCaT cells was evaluated using (**A**) MTT and (**B**) LDH assay. Cells were plated on 96-well plates and incubated overnight. Next, keratinocytes were treated with the peptide at different concentrations (5–200 μg/mL) for 3, 6, 18 h. Mean ± SD *n* = 2. ** *p* < 0.005 *** *p* < 0.001 **** *p* < 0.0001; 2way ANOVA. (**C**) Morphology of HaCaT cells was examined by confocal laser scanning microscopy. HaCaT cells were treated with CFS-conjugated DAL-PEG-DK5 (25 μg/mL) for 30 min at 37 °C and were stained with: DAPI and phalloidin for nuclear detection and actin cytoskeleton detection, respectively. Blue – DNA; red – f-actin; green-peptide conjugate; scale bar: 10 μm. Images present single slice of XY stacks.

First, we evaluated the dose-dependent toxicity of DAL-PEG-DK5 against human keratinocytes using MTT and LDH assays. We found that the peptide conjugate did not affect the metabolism of HaCaT cells at a concentration ranging from 5 to 75 μg/mL at exposure times of 3, 6, and 18 h (Figure 3A). However, higher concentrations (100 or 200 μg/mL) of DAL-PEG-DK5 reduced the number of metabolically active cells in a concentration-dependent manner (Figure 3A). By contrast, the LDH assay showed that DAL-PEG-DK5 triggered a low, but still significant, release of dehydrogenase at a concentration of 75 μg/mL (Figure 3B). Note that when human keratinocytes were incubated with 200 μg/mL of the compound, it was cytotoxic to 40% (3, 6 h) and 100% (18 h) of cells (Figure 3B). Based on the obtained data, we examined the effect of the peptide conjugate at a non-toxic dose (≤50 μg/mL) on the morphology of HaCaT cells. The data revealed that exposure of cells to the peptide did not affect their morphology (Figure 3C; bright field). Moreover, we used the CFS-labeled peptide conjugate to examine the cellular localization of DAL-PEG-DK5 to determine whether DAL-PEG-DK5 is located on the surface of HaCaT cells or internalized into the cytoplasm or nucleus. The HaCaT cells were treated with CFS-conjugated DAL-PEG-DK5 (25 μg/mL) for 30 min at 37 °C. Next, the keratinocytes were stained with DAPI and phalloidin (nuclear and actin cytoskeleton stains, respectively). As shown by confocal microscopy (Figure 3C; fluorescence), CFS-labeled DAL-PEG-DK5 appeared to localize to the cell membrane and of the cells within 30 min post-addition, without entering the nucleus.

**Figure 4:**
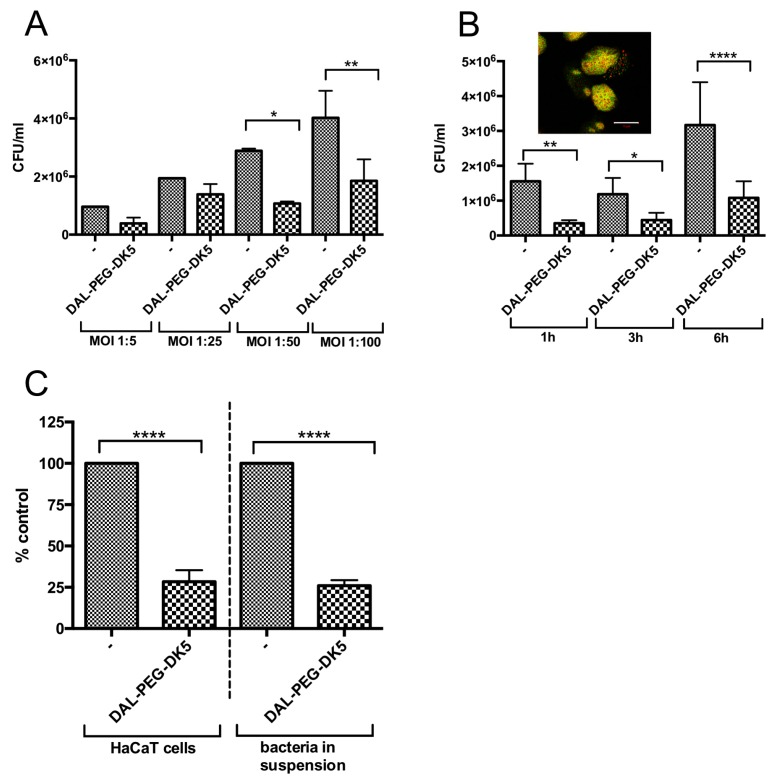
Bactericidal activity of DAL-PEG-DK5 against intracellular *S. aureus*. (**A**) USA300 survival within infected HaCaT cells. Keratinocytes were infected with USA300 (MOI 1:5, 1:25, 1:50, 1:100) for 2.5 h and treated with DAL-PEG-DK5 (50 μg/mL) for 3 h. Afterwards, keratinocytes were lysed and plated on agar plates for counting of bacteria. The number of viable bacterial cells is expressed as CFU/mL with respect to the number of intracellular bacteria in the corresponding control samples. The data shown is representative of two separate experiments performed in triplicate. Mean ± SD *n* = 2. * *p* < 0.01; ** *p* < 0.005; one-way ANOVA. (**B**) Keratinocytes were infected with USA300 (MOI 1:50) for 2.5 h and treated with DAL-PEG-DK5 (50 μg/mL) for indicated time points (1, 3, 6 h). Afterwards, keratinocytes were lysed and plated on agar plates for counting of bacteria. The number of viable bacterial cells is expressed as a CFU/mL with respect to the number of intracellular bacteria in the corresponding control samples. Mean ± SD *n* = 2. * *p* < 0.01; ** *p* < 0.005; **** *p* < 0.0001; one-way ANOVA. For confocal laser scanning microscopy HaCaT cells were infected with USA300 (MOI 1:50) for 2.5 h, then treated with DAL-PEG-DK5 (50 μg/mL) for 3 h. Afterwards cells were stained with SYTO 9 and PI. Viable *S. aureus* cells are stained green while red signals represent dead bacteria and the host cell’s nuclear DNA stained with SYTO 9 and PI. Scale bar: 10 μm. Image presents single slice of XY stacks. (**C**) Keratinocytes were infected with USA300 (MOI 1:50) for 2.5 h and treated with DAL-PEG-DK5 (50 μg/mL) for 1 h. Afterwards, keratinocytes were lysed and plated on agar plates for counting of bacteria. In parallel, MRSA (2 × 10^6^ CFU/mL) were incubated with DAL-PEG-DK5 (50 μg/mL) for 1 h and then plated on agar plates. The number of viable bacterial cells is expressed as a % of control with respect to the number of intracellular bacteria/bacteria in suspension in the corresponding control samples. Mean ± SD *n*=2. **** *p* < 0.0001; one-way ANOVA.

As we demonstrated that DAL-PEG-DK5 exerted no cytotoxic effects on keratinocytes at a dose that is strongly bactericidal against *S. aureus*, we examined the ability of DAL-PEG-DK5 to kill intracellular bacteria. For this, human keratinocytes were infected with USA300. All extracellular bacteria were removed (data not shown) by washing and treatment with lysostaphin (10 μg/mL) before adding DAL-PEG-DK5. We examined the eradication of intracellular bacteria from keratinocytes at different multiplicities of infection (MOI 1:5, 1:25, 1:50, and 1:100) and at different incubation times (1, 3, and 6 h). As depicted in Figure 4A, treatment with DAL-PEG-DK5 (50 μg/mL) reduced the intracellular USA300 load by more than 60% at an MOI 1:50 and 1:100.

Efficient elimination of the most intracellular *S. aureus* was observed at 1 h post-exposure of keratinocytes to DAL-PEG-DK5 (Figure 4B). The marked decreased in the load of intercellular *S. aureus* was also observed during prolonged (6 h) cultivation with infected cells in the presence of the peptide. To verify the vitality of intracellular bacteria, we stained cells with a LIVE/DEAD Kit (Figure 4B; insert). In concordance with the CFU (colony forming units) count, the staining results showed both live and dead intracellular staphylococci in human keratinocytes after incubation of infected cells with DAL-PEG-DK5 (50 μg/mL for 3 h). Finally, we compared the antibacterial activity of DAL-PEG-DK5 against intercellular *S. aureus* and planktonic *S. aureus*. Interestingly, we found that the ability of the peptide conjugate to kill MRSA inside HaCaT cells and in suspension was comparable (Figure 4C). The MRSA load in keratinocytes was reduced by 71.5% (compared with a 74% reduction in the number of live bacteria exposed to the peptide in suspension). Taken together, the results show that the selected temporin analogue conjugated to enkephalin exhibits potent intracellular anti-staphylococcal efficacy at a dose that is not cytotoxic to host keratinocytes.

## 3. Discussion

AMPs have gained increasing attention as novel agents for treating antibiotic-resistant *S. aureus* infections [32]. Among naturally existing host defense peptides, temporins are active against methicillin- and vancomycin-resistant staphylococci [33,34]. Here, we analyzed a set of compounds based on the sequence of an antibacterial peptide, an analogue of temporin-1CEb (DK5). This compound shows activity against MDR *S. aureus*, although it is barely described [18]. This is in contrast to other temporins such as A, B, 1Tb, CPa, CPb, 1Ga, 1Oc, 1Ola, 1Spa, and PTa [16,33,35,36,37]. To fill this gap, we performed a comparative analysis of the properties of DK5 conjugated to compounds that resemble the sequence of human peptides with known cytoprotective activity (dalargin, carnosine, and COMB1). The conjugates were first evaluated for bacteriostatic and bactericidal activity. The data revealed that the most efficient peptide conjugate was a combination of an amidated form of DK5C linked to dalargin at the C-terminus by means of PEG linker (DAL-PEG-DK5); this compound displayed marked bacteriostatic activity against all tested staphylococci.

To examine the clinical relevance of our observations, we expanded the study by examining the susceptibility of 13 isolates of MDR *S. aureus* strains to DAL-PEG-DK5. The data revealed that DAL-PEG-DK5 showed strong antibacterial activity against all tested MRSA strains. We estimated that the MIC against the majority of tested MRSA strains (90%) was 40 μg/mL (17.56 μM). This value corroborates findings by Capparelli et al., who showed that temporin A and temporin B analogues (TB-YK) exhibit antibacterial activity against clinical isolates of *S. aureus* (strains A170, A172, and 007) with MIC values ranging from 10 to 25 μg/mL [36]. Consistent with this, Ciandrini and co-workers tested the antibacterial effects of temporin A against 15 MRSA clinical isolates and found that the MIC values ranged from 4 to 16 μg/mL [16]. Moreover, temporin-PTa and its analogues display antibacterial activity against MRSA, with MIC of 0.78–3.12 μM [37]. A similar trend is seen for temporins CPa, CPb, 1Ga, 1Oc, 1Ola, and 1Spa, with MIC values ranging from 1.6 to 12.5 μM against the USA300 strain [33]. Interestingly, we noted that the Newman strain was less sensitive than MRSA strains to the tested conjugates. This can be explained by the observation published by Boyle-Vavra and co-workers, who showed that the majority of USA300 isolates were CP (capsular polysaccharide)-negative. Moreover, whole-genome sequence analysis of USA300 isolates revealed that they all carry a cap5 locus with four conserved mutations [38]. By contrast, the Newman strain shows wild-type CP5 expression [39,40]. Therefore, we suggest that the presence of CP hinders access by the peptide to the cell membrane.

We found that DAL-PEG-DK5 rapidly disrupts the staphylococcal cell membrane; at 5 min post-treatment with peptide (10 μg), there was marked permeabilization of the cell membrane, which corroborates data showing the bactericidal effects of the peptide. The efficient antibacterial activity of the DAL-PEG-DK5 conjugate may be due to the fact that it has a higher cationic charge than DK5 (+7 vs. +6, respectively) [25]. However, a α-helicity must also play an important role in this case. According to a common view about the mechanism underlying the antimicrobial activity of AMPs, formation of helices by AMPs upon binding to the membranes of target cells is a driving force behind further incorporation into the deeper layers of the membrane structure; this ultimately leads to disruption and cell leakage. For example, our previously reported circular dichroism studies show that the conjugate (DK5-PEG-DAL) was less able to form helices (in the presence of 30 mM SDS in water) than its DAL-PEG-DK5 counterpart (20% vs. 35% α-helical content, respectively) [25]. The latter (partially) explains the difference in antimicrobial activity between these two compounds. However, it should be also kept in mind that the unique amino acid sequences of these peptides define amphipathicity, charge distribution, and hydrophobicity; these factors may also play a role in the results of the present study comparing the antimicrobial activity of active DAL-PEG-DK5 with that of moderately active DK5-PEG-DAL and its inactive scrambled analogue.

Biofilms play a central role in the pathogenesis of severe staphylococcal infections (e.g., chronic wound infections or medical device-related diseases) [41,42,43]. To broaden our study, we investigated the effect of DAL-PEG-DK5 on mature biofilms formed by USA300 and showed that the conjugate is bactericidal not only against planktonic bacteria, but also against bacterial cells encrusted in the dense structure of a biofilm. Thus, we conclude that the tested peptide conjugate might be a potential new therapeutic agent for biofilm-associated infections, as revealed for analogues of temporin 1Tb [35]. However, as DAL-PEG-DK5 is cytotoxic against human keratinocytes in a dose, which is active against biofilm, therefore its topical application should be carefully considered.

The success of therapy using AMPs depends mainly on their bactericidal effects, which may be limited against intercellular pathogens. This limitation is mitigated by the amphipathic nature and high cationic charge of DAL-PEG-DK5, which enables the conjugate to enter host cells. Such a feature is deemed important for the design of therapies against chronic and relapsing staphylococcal infections, which arise as a consequence of the intracellular persistence of *S. aureus* [27,44]. Here, we show that DAL-PEG-DK5 can enter keratinocytes easily without affecting cell morphology and viability. Di Grazia and co-workers observed similar effects when studying the interaction between temporin A and B and HaCaT cells. After 30 min of incubation with keratinocytes, temporins were distributed evenly throughout the cytoplasm but did not enter the nucleus [15]. We also observed that DAL-PEG-DK5 penetrates the cell membrane and accumulates in the cytoplasm, mainly in the perinuclear area. A marked amount of the peptide was also associated with the cell membrane. Importantly, our data indicate that the peptide conjugate did not exert any cytotoxic effects against HaCaT cells at a concentration that was effective against staphylococci.

Cumulatively, these findings encouraged us to test the ability of the peptide conjugate to kill invasive intracellular MRSA. We demonstrated that after 1 h, DAL-PEG-DK5 (MIC 40 μg/mL, equal to 17.56 μM) reduced the number (by 71.5%) of viable MRSA cells inside human keratinocytes when used at 1.25 × MIC. These results agree with those of other studies on peptides with intracellular activity against *S. aureus*, which show that temporin A (at 2 × MIC (MIC 8 μM)) and temporin B (at 4 × MIC (MIC 16 μM)) killed 20% and 40% of the intracellular MRSA clinical isolate, respectively [15]. In addition, a recent report shows that another antimicrobial peptide, CPPTat-JDlys (MIC 40 μg/mL), reduced the USA300 load in keratinocytes to 20% of that of the untreated control when used at 2 × MIC [45]. Remarkably, DAL-PEG-DK5 was as effective at eradicating MRSA inside HaCaT cells as it was at killing *S. aureus* in suspension (reduction in the CFU of 71.5% vs. 74%, respectively). This appears to be due to the peptide’s ability to permeate and accumulate inside mammalian eukaryotic cells and then exert direct harmful effects against the bacterial membrane. Intracellular activity of DAL-PEG-DK5 against *S. aureus* was confirmed using infected macrophages (data not shown). We showed that temporin-1CEb is an efficient antibacterial peptide against staphylococci engulfed by human and murine macrophages, but only when combined with dalargin. Although we documented the direct bactericidal activity of DAL-PEG-DK5 and its efficient permeation of eukaryotic cells, we cannot rule out the possibility that an indirect mechanism contributes to the eradication of intracellular staphylococci from macrophages. Such a scenario is highly possible in light of the finding that DAL-PEG-DK5 exerts significant immunomodulatory effects on human macrophages [19].

In conclusion, we demonstrate that modification of temporin-1CEb to generate a peptide conjugate, DAL-PEG-DK5, makes the peptide an attractive candidate lead compound for the generation of a new agent to treat MRSA-related skin infections. Detailed in vivo studies are needed to confirm this hypothesis; however, it should be mentioned that we previously documented the stability and activity of this peptide in a murine model of sepsis [19]. The peptide inhibits planktonic growth of different clinical MRSA strains and kills them in the biofilm. DAL-PEG-DK5 acts by disrupting the integrity of the bacterial cell membrane without damaging keratinocytes. Such a unique mechanism is probably the consequence of combining temporin with dalargin, which is known for its cytoprotective properties towards human epithelial cells, including keratinocytes [25,46]. Taken together, the data presented herein indicate that the DAL-PEG-DK5 conjugate is a candidate treatment for skin infections caused by MRSA.

## 4. Materials and Methods

### 4.1. Reagents

Gentamicin, vancomycin, linezolid, and lysostaphin were from Sigma-Aldrich (St. Louis, MO, USA). FBS, DMEM, RPMI 1640, calcium and magnesium free phosphate-buffered saline (PBS without Ca^2+^ and Mg^2+^), and penicillin-streptomycin (PEST) were obtained from Gibco (Life Technologies, Paisley, UK). CytoTox96 non-radioactive cytotoxicity assay kit was obtained from Promega (Promega, Madison, WI, USA). Sytox green and LIVE/DEAD BacLight Bacterial Viability kit were purchased from ThermoFisher Scientific (Invitrogen, ThermoFisher Scientific, Eugene, OR, USA).

### 4.2. Peptides Synthesis and Purification

All compounds were synthesized manually by means of the solid phase method applying Fmoc (fluorenyl-9methoxycarbonyl) chemistry under the standard conditions. S RAM (substitution 0.25 meq/g, RAPP Polymere, Tubingen, Germany) resins were used as solid support. The peptide chain was elongated by means of Fmoc-protected amino acids (3 equiv) using HOBt (N-hydroxybenzotriazole)/HBTU (N,N,N′,N′-Tetramethyl-O-(1H-benzotriazol-1-yl) uronium hexafluorophosphate) (3 equiv) as coupling reagents in the presence of DIPEA (N,N-Diisopropylethylamine) (6 equiv). The completeness of each coupling was monitored by the Kaiser test. The Fmoc-protection after each step of coupling was removed with 20% piperidine in dimethylformamide (DMF). For fluorescent labeling, N-terminal fluorescein moiety was introduced to the sequence via its succinimide derivative. Cleavage of the peptides from the resin was achieved using a TFA/phenol/triisopropylsilane/H2O mixture (88:5:2:5, *v*/*v*).

The synthesized compounds were purified by reverse phase high performance chromatography (RP-HPLC) on Waters system (Phenomenex Jupiter 4 µ Proteo 90 Å column, 250 × 10 mm). The linear gradient from 10% to 80% B within 60 min (A: 0.1% TFA in water; B: 80% acetonitrile in A) with a flow rate 5 mL/min was employed. The homogeneity of the final fractions of peptides were analyzed on Shimadzu HPLC System (Shimadzu Europe GmbH, Duisburg, Germany) equipped with Phenomenex Jupiter 4 µ Proteo 90 Å column, 250 × 4.60 mm column. Mass spectra of the synthesized peptides were recorded using a Biflex III MALDI TOF mass spectrometry (Bruker, Mannheim, Germany) with α-cyano-4-hydroxy-cinnamic acid (CCA) or 2,5-dihydroxybenzoic acid (DHB) used as the matrix.

### 4.3. Cell Culture

A well-established line of human immortalized keratinocytes (HaCaT cell line) were obtained from American Type Culture Collection (ATCC, Manassas, VA, USA) and cultured in DMEM (Gibco, Life Technologies, Paisley, UK) supplemented with 10% heat-inactivated fetal bovine serum (FBS) and PEST (100 U/mL penicillin and 100 U/mL streptomycin) at 37 °C in humidified 5% CO_2_ atmosphere. Cells were passaged every 4–5 days.

### 4.4. Microorganisms

The staphylococcus strains used in this study, listed in Table 4, were stored in tryptic soy broth (TSB, Sigma Aldrich, St. Louis, MO, USA) containing glycerol (50% *v*/*v*) at −80 °C. Cultures were inoculated from stocks into 10 mL media. Strains were grown overnight under constant rotation (180 rpm) to mid-logarithmic growth phase at 37 °C, centrifuged at 5000× *g* for 5 min, washed in PBS, and resuspended in PBS to the desired OD (600 nm).

### 4.5. Cell Viability Test

The viability of HaCaT cells was examined by a 3-(4,5-dimethylthiazol-2-yl)-2,5-diphenyltetrazolium bromide (MTT, Sigma-Aldrich) reduction assay. In brief, cells were incubated with 10% (*v*/*v*) of a 5 mg/mL MTT solution for 1 to 2 h at 37 °C until purple precipitate is visible and acidified isopropanol was added followed by measuring of the absorbance at a wavelength of 570 nm.

To assess the effect of peptides on the integrity of the plasma membrane, the LDH release assay was performed using the CytoTox96 nonradioactive cytotoxicity assay kit (Promega, Madison, WI, USA) according to the manufacturer’s instructions. Cytotoxicity was calculated with the formula: % cytotoxicity = 100 × (experimental LDH release/ maximum LDH release), where maximum LDH release is after lysis solution addition (Triton X-100). Relative amounts of LDH release were measured (absorbance at 490 nm) using plate reader SpectraMax (Molecular Device, Wokingham, UK). All assays were performed in triplicate.

### 4.6. Antimicrobial Activity

Antimicrobial activity of peptides was analyzed through determination of MIC parameter according to the standard microdilution technique performed on 96-well plates. Mueller-Hinton broth (MHB, Sigma Aldrich, St. Louis, MO, USA) was used as the working medium for all bacterial strains. Briefly, inoculum was prepared from freshly grown cultures of bacteria being at their exponential phase of growth. Each well of 96-well plates containing 100 µL of serially diluted peptides in PBS at desired concentration was inoculated with 100 µL of 10^5^ CFU/mL of bacterial suspension. Then plates were incubated overnight 37 °C and absorbance was read at 600 nm after 24 h. Wells without peptide were treated as the positive control, while uninoculated MHB was defined as the negative control. All measurements were run in triplicates. MIC was defined as the minimal concentration that completely inhibits growth of microorganisms was performed according to Clinical and Laboratory Standards Institute (CLSI).

### 4.7. Efficacy of Peptides on S. aureus Biofilms

The efficacy of peptides to disrupt mature biofilms was followed as described before [48]. Briefly, USA300 strain grown overnight was diluted 1:100 in TSB + 1% glucose and incubated in 96-well plates at 37 °C for 24 h. After removing media, wells were rinsed with PBS to remove planktonic bacteria before re-filling wells with fresh MHB. Peptide conjugates were added at desired concentrations and plates were incubated at 37 °C for 24 h. After incubation, wells were washed and biofilms were stained with 0.5% (*w*/*v*) crystal violet for 30 min. The dye was solubilized with ethanol (95%) and the OD of biofilms was measured.

### 4.8. Sytox Green Uptake Analysis

The USA300 strain was grown to mid-logarithmic phase at 37 °C, washed, and resuspended in 10 mM sodium phosphate buffer (pH 7.2) Bacterial cells were then incubated with 1 µM Sytox green for 15 min in the dark. After the addition of desired concentration of peptide conjugate (5–100 µg/mL), the time-dependent increases in fluorescence caused by the binding of the cationic dye to intracellular DNA were monitored (excitation at 485 nm and emission at 520 nm) [49].

### 4.9. Assessment of Bacterial Viability by Using the LIVE/ DEAD BacLight KIT

USA300 cells were collected from overnight cultures, at the end of the exponential growth phase and the beginning of the stationary phase, washed and suspended at 2 × 10^7^ bacteria/mL and staphylococci were treated with DAL-PEG-DK5 (0.5–50 µg/mL) for 3 h. In order to obtain a standard curve, five different proportions of live and dead bacteria were mixed prior to staining. After the incubation, the 100 µL of bacterial suspensions were transferred to flat bottom black 96-well microtitration plate. Staining solution containing SYTO9 and PI (100 µL) prepared according to manufacturer’s instructions was then mixed with bacterial suspensions. Samples were incubated at room temperature in the dark for 15 min and fluorescence intensity was measured with FlexStation3 Multimode Microplate Reader using a 485 nm excitation filter (for both SYTO9 and PI) and a 530 nm (SYTO9 emission wavelength) and 630 nm (PI emission wavelength) emission filter. The data were analyzed by dividing the fluorescence intensity of the stained bacterial suspensions at green emission by the cell fluorescence intensity of red fluorescence. All samples were prepared in triplicates.

### 4.10. Antibacterial Efficacy of Peptides Against Intracellular S. aureus

Infection of HaCaT keratinocytes was conducted as previously described [50]. Human keratinocytes were grown in standard medium in 12 (5 × 10^5^ cells) or 24-well (3 × 10^5^ cells) tissue culture plates. Following incubation, the cells were infected with USA300 at different multiplicity of infection (MOI; 1:5, 1:25, 1:50, 1:100) in DMEM supplemented with 10% FBS, without PEST for 2.5 h. After infection, the cells were washed with ice cold PBS and further incubated for 30 min with lysostaphin (10 μg/mL) to kill extracellular bacteria. DAL-PEG-DK5 was diluted in culture media to 50 μg/mL and added to the cells for an additional 1, 3, and 6 h. The medium alone was used as a negative control. After incubation, media were aspirated and human keratinocytes were washed twice with PBS to remove any residual peptide. Then HaCaT cells were lysed with ice cold H_2_O. The cell lysates were serially diluted and plated on TSA plates.

### 4.11. Confocal Microscopy

HaCaT keratinocytes were seeded on coverslips for 24 h in DMEM supplemented with 10% FBS at 37 °C in humidified 5% CO_2_ atmosphere. After 24 h, cells were washed with PBS and treated with FITC conjugated DAL-PEG-DK5 (25 μg/mL) for 30 min at 37 °C. Then cells were washed with cold PBS and fixed with 3.7% formaldehyde for 15 min at RT. Afterwards, HaCaT keratinocytes were stained with DAPI and phalloidin for nuclear and actin cytoskeleton detection, respectively. The coverslips were placed on glass slide with mounting media and visualized using Zeiss LSM 880 confocal system.

To distinguish vivid bacteria with the intact cell membrane from dead bacteria with the compromised membrane, the LIVE/DEAD BacLight Bacterial Viability kit was used. SYTO 9 and PI were mixed in proper ratios, and gave fluorescence signals indicative of alive or dead bacteria. Before staining HaCaT cells were permeabilized with 0.2% Triton X-100 to allow PI to bind to the dead bacteria and keratinocyte DNA. Confocal laser scanning microscopy (CSLM) was carried out using Zeiss LSM 880 confocal system equipped with 100× oil immersion objectives and acquired images analyzed in Zeiss ZEN Microscope Software.

### 4.12. Statistical Analysis

Statistical comparisons were performed with Prism 6.0 software (GraphPad Software, Inc., San Diego, CA, USA), using one- or two-way ANOVA test for multiple comparisons. *p* value < 0.05 was considered to be significant.

## Figures and Tables

**Table 1:** MIC of tested peptides conjugates against *S. aureus* strains and *S. epidermidis*.

Staphylococcal strain	Strain ID	MIC [μg/mL]			
		DAL-PEG-DK5	CAR-PEG-DK5	CAR3-PEG-DK5	COMB1-PEG-DK5
*S. aureus*	USA300	40	>190	>190	>190
	Newman	140	>190	>190	>190
	ATCC 25923	40	>190	>190	>190
*S. epidermidis*	ATCC 12228	60	40	110	>190

**Table 2:** MIC of native peptides, their peptides conjugates, and SCR against methicillin resistant *S. aureus* (MRSA) isolate.

USA300	MIC [μg/mL]
DK5	>190
DAL	>190
DK5-PEG-DAL	160
DAL-PEG-DK5	40
SCR	>190

**Table 3:** MIC of DAL-PEG-DK5 against methicillin resistant *S. aureus* (MRSA) clinical isolates.

MRSA strain ID	MIC [μg/mL]		
	DAL-PEG-DK5	Vancomycin	Linezolid
56A1	40	-	-
52B	40	0.5	2
1694	70	-	-
2492	40	-	-
2706	40	-	-
2872cv	40	-	-
3417	40	-	-
4187	40	-	-
6674	40	-	-
7219	30	-	-
7501	40	-	-
7569	40	-	-
7718	50	-	-
USA300	40	1	1

- not determined.

**Table 4:** Staphylococcus strains used in this study.

Staphylococcus Strains	Relevant Properties	Source
*S. aureus*		
USA300	Wilde type strain	L.N. Shaw [47]
ATCC 25923	Clinical isolate	ATCC
Newman	Wilde type laboratory strain	T.J. Foster
56A1	Clinical isolate	*
52B	Clinical isolate	*
1694	Clinical isolate	*
2492	Clinical isolate	*
2706	Clinical isolate	*
2872cv	Clinical isolate	*
3417	Clinical isolate	*
4187	Clinical isolate	*
6674	Clinical isolate	*
7219	Clinical isolate	*
7501	Clinical isolate	*
7569	Clinical isolate	*
7718	Clinical isolate	*
*S. epidermidis*		
ATCC 12228	Wilde type strain	ATCC

* The clinical strains were collected from nonrelated patients admitted to the Stefan Zeromski Specialist Muncipal Hospital in Krakow, Poland.

## Supplementary Materials

Supplementary materials can be found at https://www.mdpi.com/1422-0067/20/19/4761/s1.

## Abbreviations

AMPs	Antimicrobial peptides
DMEM	Dulbecco’s Modified Eagle’s Medium
CFU	Colony forming units
FBS	Fetal bovine serum
MIC	Minimal inhibitory concentration
MOI	Multiplicity of infection
OD	Optical density
PBS	Phosphate buffered saline
PI	Propidium iodide
SCR	Scramble peptide
