# Supplementary material for: The Bactericidal Activity of Temporin Analogues Against Methicillin Resistant Staphylococcus aureus
Source: Int J Mol Sci. 2019 Sep 25;20(19):4761. doi: 10.3390/ijms20194761 (PMC6801822; doi:10.3390/ijms20194761)
Supplement: Supplementary file 1 [file ijms-20-04761-s001.pdf]

Table S1. Primary structures of the peptides.

Compound	Primary sequence
DK5	IKKILS**k**IKKLL-NH_2
DAL	Y**a**GFLR
CAR	ßAH
CAR3	ßAH ßAH ßAH
COMB1	EGLEPG
DK5-PEG-DAL	IKKILS**k**IKKLL-**PEG**-Y**a**GFLR
DAL-PEG-DK5	Y**a**GFLR-**PEG**-IKKILS**k**IKKLL-NH_2
CAR-PEG-DK5	ßAH-**PEG**-IKKILS**k**IKKLL-NH_2
CAR3-PEG-DK5	ßAHßAHßAH-**PEG**-IKKILS**k**IKKLL-NH_2
COMB1-PEG-DK5	EGLEPG-**PEG**-IKKILS**k**IKKLL-NH_2

D-analogues of the amino acids are marked as small bold letters; ßA refers to ß-Ala amino acid residue.

Table S2. Characterization of methicillin resistant *S. aureus* (MRSA) clinical isolates.

MRSA strain ID	Source	Macrolide resistance phenotype	Other antibiotic resistance phenotype
56A1	skin	cMLS$_B$	P, C, CL, DO*, G, TOB, K
52B	skin	lack of resistance	P
1694	blood	cMLS$_B$	P, TE, CIP, CN, AK, TOB, DO, K
2492	blood	cMLS$_B$	P, TE, CIP, AK*, TOB, K
2706	blood	cMLS$_B$	P, CIP, TOB, K
2872cv	blood	cMLS$_B$	P, TE, CIP, CN, AK, TOB, DO, K
3417	blood	cMLS$_B$	P, K*
4187	blood	lack of resistance	P
6674	blood	cMLS$_B$	P, TE, CIP, CN, AK, TOB, DO, K
7219	blood	cMLS$_B$	P, TE, CIP, CN, AK, TOB, DO, K
7501	blood	iMLS$_B$	P, CIP, TOB, K, DA
7569	blood	iMLS$_B$	P, CIP, AK*, TOB, K, DA
7718	blood	cMLS$_B$	P, CIP, TOB, K

MLS$_B$, resistance to macrolides, lincosamides and streptogramins B; the prefix letter refers to the constitutive expression of this phenotype (*cMLS$_B$*) or inducible expression of the phenotype (*iMLS$_B$*); *AK*, amikacin; *C*, chloramphenicol; *CIP*, ciprofloxacin; *CL*, clindamycin; *CN*, cephalexin; *DA*, dalfopristin; *DO*, doxycycline; *G*, gentamycin; K, kanamycin; *P*, penicillin; *TE*, tetracycline; *TOB*, tobramycin; *XXX**, intermediate resistance.
